# Healthcare-associated infections and antimicrobial resistance in Canadian acute care hospitals, 2019–2023

**DOI:** 10.14745/ccdr.v51i67a04

**Published:** 2025-07-01

**Authors:** Canadian Nosocomial Infection Surveillance Program

**Affiliations:** 1 Centre for Communicable Diseases and Infection Control, Public Health Agency of Canada, Ottawa, ON

**Keywords:** healthcare-associated infections, community-associated infections, antimicrobial resistance, surveillance, *Clostridioides difficile* infection, methicillin-resistant *Staphylococcus aureus*, vancomycin-resistant Enterococcus, carbapenemase-producing *Enterobacterales*, *Escherichia coli*, *Candida auris*, Canadian Nosocomial Infection Surveillance Program

## Abstract

**Background:**

Healthcare-associated infections (HAIs) and antimicrobial resistance (AMR) continue to contribute to excess morbidity and mortality among Canadians.

**Objective:**

This report describes epidemiologic and laboratory characteristics and trends of HAIs and AMR, 2019–2023, using surveillance and laboratory data submitted by hospitals to the Canadian Nosocomial Infection Surveillance Program (CNISP) and by provincial and territorial laboratories to the National Microbiology Laboratory.

**Methods:**

Data was collected from 109 Canadian sentinel acute care hospitals between January 1, 2019 and December 31, 2023, for *Clostridioides difficile* infections (CDI), methicillin-resistant *Staphylococcus aureus* (MRSA) bloodstream infections (BSIs), vancomycin-resistant *Enterococcus* (VRE) BSIs (specifically *Enterococcus faecalis* and *Enterococcus faecium*), carbapenemase-producing *Enterobacterales* (CPE) and carbapenemase-producing *Acinetobacter baumannii* (CPA) infections and colonizations and *Candida auris* (*C. auris*). Trend analysis for case counts, incidence rates (rates), outcomes, molecular characterization and AMR profiles are presented.

**Results:**

Rates remained relatively stable for CDI (range: 4.90–5.35 infections per 10,000 patient days) and MRSA BSI (range: 1.00–1.16 infections per 10,000 patient days) and increased significantly for VRE BSIs (range: 0.30–0.37 infections per 10,000 patient days). Infection rates for CPE remained low compared to other HAIs but doubled non-significantly (rates: 0.08–0.16), CPA counts remained very low (n=4 cases) and *C. auris* isolates remained low (n=36 isolates).

**Conclusion:**

The incidence of MRSA BSIs and CDI remained stable and VRE BSIs and CPE infections increased in the Canadian acute care hospitals participating in CNISP. Few *C. auris* isolates were identified. Reporting standardized surveillance data to inform the application of infection prevention and control practices in acute care hospitals is critical to help decrease the burden of HAIs and AMR in Canada.

## Introduction

Healthcare-associated infections (HAI) represent one of the most common adverse events experienced by patients in acute care settings globally ((1)). In addition to increasing morbidity and mortality, they are associated with longer lengths of stay in hospitals and higher costs of care ((1)). In Canada, a point prevalence survey conducted in 2017 estimated that the prevalence of patients with at least one HAI was 7.9% ((2)). The prevalence of HAIs between 2015–2018 has been estimated to be at 3.2% in the United States (US), 6.5% in Europe and 9.9% in Australia (and is likely two-fold greater in developing countries) ((1,3–5)). In Europe, the cumulative healthcare burden of six HAIs (urinary tract infection, pneumonia, surgical site infection, *Clostridioides difficile* infections [CDIs], bloodstream infections [BSIs] and neonatal sepsis) was greater than the burden of 32 other communicable diseases combined, including influenza and tuberculosis ((6)). Importantly, a large proportion of HAIs are preventable and evidence from the US showed that advancements in care and infection prevention and control can decrease HAI rates over time ((4)).

Many of the microorganisms that cause HAIs have a propensity for antimicrobial resistance (AMR) and growing rates of resistance threaten to undermine efforts to reduce HAI rates ((6)). Infection with a resistant organism is associated with an 84.4% increased risk of death and in 2019, bacterial AMR was associated with approximately five million deaths globally ((7,8)). Canadian data have shown that CDI is associated with a longer length of hospital stay, higher all-cause mortality and an average excess cost of $11,056 per patient ((9)). Other data from Canada and abroad have shown that *Staphylococcus aureus* (MRSA) BSIs contributed to significant morbidity and mortality, prolonged hospital stays and increased healthcare costs for hospitalized patients ((10–13)). The rate of AMR is predicted to reach 40% by 2050. In this situation, it is forecasted that 13,700 Canadians could die each year from resistant infections and the overall annual impact to Canada’s GDP would be $21 billion ((14)). Moreover, emerging resistant pathogens, such as *Candida auris,* have necessitated enhanced surveillance and changes to existing infection prevention and control protocols ((15)). Coordinated global public health action, surveillance, improved antibiotic stewardship, infection prevention and control and public awareness are crucial to identify patterns of antimicrobial resistance and prevent and control emerging infections ((16)).

In Canada, the Public Health Agency of Canada (PHAC) collects national data on various HAIs and AMR through the Canadian Nosocomial Infection Surveillance Program (CNISP). Established in 1994, CNISP is a collaboration between the PHAC, the Association of Medical Microbiology and Infectious Disease Canada and sentinel hospitals from across Canada. The goal of CNISP is to facilitate and inform the prevention, control and reduction of HAIs and antimicrobial resistant organisms in Canadian acute care hospitals through active surveillance and reporting.

In line with the World Health Organization’s core components of infection prevention and control ((17)), CNISP performs consistent, standardized surveillance to reliably estimate HAI burden, establish benchmark rates for national and international comparison, identify potential risk factors and assess and inform specific interventions to improve patient health outcomes. Data provided by CNISP directly support the collaborative goals outlined in the *Pan-Canadian Action Plan on Antimicrobial Resistance* ((16)).

In this report, we describe the most recent HAI and AMR surveillance data collected from CNISP participating hospitals between 2019 and 2023.

## Methods

### Design

The Canadian Nosocomial Infection Surveillance Program conducts prospective, sentinel surveillance for HAIs (including antimicrobial resistant organisms) ((18)).

### Case definitions

Standardized case definitions for healthcare-associated (HA) and community-associated (CA) infections were used. Refer to **Appendix A** for full-case definitions.

### Data sources

Between January 1, 2019 and December 31, 2023, participating hospitals submitted epidemiologic data and isolates for cases meeting the respective case definitions for CDIs, MRSA BSIs, vancomycin-resistant *Enterococcus* (VRE) BSIs (specifically *Enterococcus faecalis* and *Enterococcus faecium*) and carbapenemase-producing *Enterobacterales* (CPE) and carbapenemase-producing *Acinetobacter baumannii* (CPA) (infections or colonizations). *C. auris* isolates (infections or colonizations) were identified by provincial and territorial laboratories and participating hospital laboratories. In 2023, 109 hospitals in 10 provinces and one territory participated in HAI surveillance and are further described in **Table 1**. Hospital participation varied by surveillance project and year (**Appendix B,** supplemental figures and tables are available upon request from the author). In 2023, CNISP HAI surveillance, patient admissions were categorized according to hospital bed size; small (1–200 beds, n=56 sites, 51%), medium (201–499 beds, n=34 sites, 31%) or large (500 or more beds, n=19 sites, 17%). Hospital participation also varied by region: Western (British Columbia, Alberta, Saskatchewan and Manitoba, n=44 sites, 40%), Central (Ontario and Québec, n=38 sites, 35%), Eastern (Nova Scotia, New Brunswick, Prince Edward Island and Newfoundland and Labrador, n=26 sites, 24%) and Northern (Yukon, Northwest Territories and Nunavut, n=1, 0.9%) (Table 1).

**Table 1:** Summary of hospitals participating in the Canadian Nosocomial Infection Surveillance Program, by region, 2023

Details of participating hospitals	Western^a^	Central^b^	Eastern^c^	Northern^d^	Total
Total number of hospitals	44	38	26	1	109
Adult^e^	23	21	16	0	60
Mixed^f^	17	13	9	1	40
Paediatric^g^	4	4	1	0	9
Small (1–200 beds)	20	13	22	1	56
Medium (201–499 beds)	15	16	3	0	34
Large (500 or more beds)	9	9	1	0	19
Total number of beds	12,340	13,164	3,197	25	28,726
Total number of admissions	469,988	558,545	110,607	2,093	1,141,233
Total number of patient days	3,691,976	4,180,827	1,031,841	6,952	8,911,596

^a^ Western refers to British Columbia, Alberta, Saskatchewan and Manitoba

^b^ Central refers to Ontario and Québec

^c^ Eastern refers to Nova Scotia, New Brunswick, Prince Edward Island and Newfoundland and Labrador

^d^ Northern refers to Yukon, Northwest Territories and Nunavut

^e^ Eleven hospitals classified as “adult” had a neonatal intensive care unit

^f^ Mixed hospitals provide both adult and paediatric care

^g^ Paediatric standalone hospitals excluding mixed facilities with women’s and obstetric wards

Epidemiologic (demographic, clinical and outcomes) and denominator data (patient days and patient admissions) were collected and submitted by participating hospitals through the Canadian Network for Public Health Intelligence, a secure online data platform.

Reviews of standardized protocols and case definitions are conducted annually by established infectious disease expert working groups; training for data submission is provided to participating CNISP hospital staff as required. Data quality for surveillance projects is periodically evaluated; additional details on the methodology have been published previously ((19,20)).

### Laboratory data

All patient-linked laboratory isolates (stool samples for CDI cases) were sent to the PHAC’s National Microbiology Laboratory for molecular characterization and antimicrobial susceptibility testing. Isolates for MRSA BSIs, VRE BSIs, CPE/CPA (infections or colonizations), *C. auris* (infections/colonizations) and paediatric CDIs were submitted year-round. Adult CDI isolates were submitted annually during a targeted two-month period (March 1 to April 30).

### Statistical analysis

Rates of HAI were calculated by dividing the total number of cases identified in patients admitted to CNISP participating hospitals by the total number of patient admissions (multiplied by 1,000) or patient days (multiplied by 10,000). Due to low case numbers, rates for *C. auris* were not calculated. The HAI rates are reported nationally and by region. Due to the low number of CA-VRE BSI cases reported each year, stratified rates as well as mortality rates and laboratory results for CA-VRE BSIs were not included in this report. Sites that were unable to provide case data were excluded from rate calculations and missing denominator data were estimated using their previous years reported data, where applicable. Missing epidemiological and molecular data were excluded from analysis. The Mann-Kendall test was used to assess trends in rates over time. The chi-square test for trend was used to analyze trends in proportions over time. The chi-square test was used to compare two categorical variables, while the t-test was used to compare differences between groups. Significance testing was two-tailed and differences were considered significant at *p*≤0.05. The stability of rates over time indicates that there was no statistically significant trend observed. Where available, all-cause mortality were reported for HAIs. All-cause mortality rate was defined as the number of deaths per 100 HAI cases 30 days following positive culture.

## Results

### *Clostridioides difficile* infection

Between 2019 and 2023, overall CDI rates remained stable, ranging from 4.90 to 5.35 infections per 10,000 patient days. Rates initially rose from 2019 to 2020, then decreased from 2020 to 2022, before rising again in 2023. However, no significant trend was observed (*p*=1.0) (**Table 2**). The median age among CDI patients was 69 years (IQR: 55–79), with males and females each representing 50% of the total cases (Appendix B).

**Table 2:** *Clostridioides difficile* infection data, Canada, 2019–2023^a^

*C. difficile* infection data	Number of infections and incidence rates (per year)				
	2019	2020	2021	2022	2023
**All cases**					
Number of *C. difficile* infection cases	3,600	3,650	3,640	3,878	4,453
Rate per 1,000 patient admissions	3.69	4.10	3.93	4.13	4.06
Rate per 10,000 patient days	4.90	5.35	5.07	4.97	5.05
Number of reporting hospitals	73	82	82	82	98
**All-cause mortality rate**					
Number of deaths	63	54	66	64	74
All cause mortality rate per 100 cases (%)^b^	8.5	9	8.8	8.9	8.2
**HA-CDI**					
Number of HA-CDI cases	2,662	2,625	2,571	2,819	3,142
Rate per 1,000 patient admissions	2.73	2.95	2.78	3.00	2.86
Rate per 10,000 patient days	3.62	3.85	3.58	3.62	3.56
Number of reporting hospitals	73	82	82	82	98
**All-cause mortality rate**					
Number of deaths	47	39	50	54	58
All cause mortality rate per 100 cases (%)^b^	8.2	8.7	9.2	9.9	8.7
**CA-CDI**					
Number of CA-CDI cases	938	1,025	1069	1,059	1,311
Rate per 1,000 patient admissions	1.17	1.38	1.38	1.35	1.42
Rate per 10,000 patient days	1.57	1.83	1.81	1.65	1.77
Number of reporting hospitals	62	71	71	71	87
**All-cause mortality rate**					
Number of deaths	16	15	16	10	16
All cause mortality rate per 100 cases (%)^b^	9.4	9.6	7.4	5.8	6.3

Abbreviations: *C. difficile*, *Clostridioides difficile*; CA, community-associated; CDI, *Clostridioides difficile* infections; HA, healthcare-associated

^a^ There was no resistance to tigecycline, vancomycin or metronidazole in *C. difficile* isolates submitted to the National Microbiology Laboratory 2019–2023

^b^ Mortality data are collected during the two-month period (March and April of each year) for adults (aged 18 years and older) and year-round for children (aged one year to younger than 18 years old). Among paediatric patients, there was no death attributable to healthcare-associated *C. difficile* infection

**Source of infection:** Stratified by the source of infection, from 2019 to 2023, the incidence of HA-CDI showed little change from 3.62 to 3.56 infections per 10,000 patient days (*p*=0.31) (Table 2). The CA-CDI rates increased from 1.17 to 1.42 infections per 1,000 patient admissions when comparing 2019 to 2023 rates; however, this trend was not significant (*p*=0.32) (Table 2).

Regionally, HA-CDI rates have fluctuated across all regions with non-significant changes observed between 2019 and 2023. Specifically, the Western region had an overall decrease from 3.34 to 3.13 infections per 10,000 patient days (*p*=0.46), the Central region remained stable (3.40–3.77 infections per 10,000 patient days (*p*=0.61), the Eastern region had a steady rate increase from 2019 to 2021 (2.90–3.58 infections per 10,000 patient days, *p*=0.09) with a significant drop from 2022 to 2023 (3.58–3.02 infections per 10,000 patient days, *p*=0.05). For CA-CDI, rates per 1,000 patient admissions remain highest in the Central region from 2019 and 2023 (range: 1.39–1.65), followed by Western (range: 0.99–1.58) and Eastern (range: 0.68–1.05) (Appendix B).

**Hospital types:** The HA-CDI rates per 10,000 patient days were consistently higher in adult (range: 3.62–3.84, *p*≤0.005) and paediatric hospitals (range: 3.09–3.61, *p*≤0.005), with lower rates observed in mixed hospitals (range: 2.57–3.06). The CA-CDI rates per 1,000 patient admissions were higher in adult (range: 1.55–1.77, *p*≤0.005) and mixed hospital (range: 1.26–1.61), with lower rates observed in paediatric hospitals (range: 0.57–1.15, *p*≤0.005) between 2019 and 2023 (Appendix B). Stratified by hospital size, rates of HA-CDI were generally highest among large (range: 3.28–3.87), followed by medium (range: 3.15–3.55) and small size hospitals (range: 2.43–2.84). Rates of CA-CDI per 1,000 patient admissions were similar for large (range: 1.23–1.69) and medium sized hospitals (range: 1.22–1.47), and lower for small sized hospitals (range: 0.69–1.19) (Appendix B).

**30-day all-cause mortality:** Overall 30 day all-cause CDI mortality remained stable over time (range: 8.2–9.0 deaths per 100 cases) (*p*=0.81) between 2019 and 2023 (Table 2). In 2023, 30-day all-cause mortality was significantly higher for HA-CDI (8.7%) compared to CA-CDI (6.3%) (*p*=0.02).

**Antimicrobial resistance:** From 2019 to 2023, 27.1% (n=656/2,424) of CDI isolates were resistant to one or more tested antimicrobials. The proportion of *C. difficile* isolates resistant to moxifloxacin significantly decreased (*p*=0.002) between 2019 (11.6%, n=66/568) and 2023 (6.3%, n=32/506) (**Table 3**). Since 2019, moxifloxacin resistance decreased non-significantly among HA-CDI isolates (5.2%, *p*=0.22) while a smaller non-significant decrease was observed among CA-CDI (5.4%, *p*=0.31) (Appendix B). There was no resistance to metronidazole, vancomycin, or tigecycline for all *C. difficile* isolates tested.

**Table 3:** *Clostridioides difficile* antimicrobial resistance data, Canada, 2019–2023^a,b^

Antibiotic	Number of isolates and % resistance (per year)									
	2019		2020		2021		2022		2023	
n	%	n	%	n	%	n	%	n	%
Clindamycin	221	38.9	62	17.0	67	12.4	101	22.8	66	13.0
Moxifloxacin	66	11.6	24	6.6	49	9.0	31	7.0	32	6.3
Rifampin	6	1.1	3	0.8	9	1.7	4	0.9	4	0.8
Total number of isolates tested^c^	568	N/A	365	N/A	542	N/A	443	N/A	506	N/A

Abbreviation: N/A, not applicable

^a^ *Clostridioides difficile* infection isolates are collected for resistance testing during the two-month period (March and April of each year) for adults (aged 18 years and older) and year-round for children (aged one year to younger than 18 years old) from admitted patients only

^b^ There was no resistance to tigecycline, vancomycin, or metronidazole in *C. difficile* isolates submitted to the National Microbiology Laboratory 2019–2023

^c^ Total reflects the number of isolates tested for each of the antibiotics listed above

**Molecular typing:** From 2019 to 2023, the top five most prevalent ribotypes of isolates from HA-CDI defined cases were 106, 014, 020, 002 and 027, with overall prevalences of 15.3%, 9.0%, 6.9%, 6.0% and 5.7%, respectively, while the top five ribotypes of isolates from CA-CDI were 106, 014, 020, 015 and 002, with overall prevalences of 15.2%, 7.9%, 7.4% 5.8% and 5.2%. From 2019 to 2023, the prevalence of RT027 associated with NAP1 decreased from 7.3% to 3.3% and from 3.1% to 2.1%, in HA and CA-CDI populations respectively (Appendix B).

### Methicillin-resistant *Staphylococcus aureus* bloodstream infections

Between 2019 and 2023, overall MRSA BSI rates remained stable, ranging from 1.00 to 1.16 infections per 10,000 patient days. Rates peaked in 2020 (n=1.16) and were lowest in 2022 (n=1.00); however, no significant trend was observed (*p*=0.462) (**Table 4**). The median age among MRSA BSI patients was 55 years (IQR: 39–70), with women accounting for 38% of cases (Appendix B).

**Table 4:** Methicillin-resistant *Staphylococcus aureus* bloodstream infections data, Canada, 2019–2023

MRSA BSI data	Year				
	2019	2020	2021	2022	2023
**All cases**					
Number of MRSA BSIs	881	868	875	841	913
Rate per 1,000 patient admissions	0.85	0.88	0.85	0.81	0.90
Rate per 10,000 patient days	1.14	1.16	1.12	1.00	1.13
Number of reporting hospitals	69	81	81	81	76
**All-cause mortality rate^a^**					
Number of deaths	144	152	166	167	174
All-cause mortality rate per 100 cases	16.3	17.5	18.9	19.9	19.1
**HA-MRSA BSI**					
Number of HA-MRSA BSIs	364	323	351	352	377
Rate per 1,000 patient admissions	0.35	0.33	0.34	0.34	0.37
Rate per 10,000 patient days	0.47	0.43	0.45	0.42	0.47
Number of reporting hospitals	69	81	81	81	76
**All-cause mortality rate^a^**					
Number of deaths	74	65	88	84	93
All-cause mortality rate per 100 cases	20.3	20.1	25.0	23.9	24.7
**CA-MRSA BSI**					
Number of CA-MRSA BSIs	450	480	471	453	527
Rate per 1,000 patient admissions	0.43	0.49	0.46	0.44	0.52
Rate per 10,000 patient days	0.59	0.65	0.61	0.55	0.67
Number of reporting hospitals	68	80	80	80	75
**All-cause mortality rate^a^**					
Number of deaths	61	76	71	79	80
All-cause mortality rate per 100 cases	13.6	15.8	15.1	17.4	15.2

Abbreviations: CA, community-associated; HA, healthcare-associated; MRSA BSI, methicillin-resistant *Staphylococcus aureus* bloodstream infection

^a^ Based on the number of cases with associated 30-day outcome data

**Source of infection:** The CA-MRSA BSI rates increased slightly, from 0.59 in 2019 to 0.67 infections per 10,000 patient days in 2023, though the trend was not significant (*p*=0.81). Healthcare-associated-MRSA BSI rates remained stable (range: 0.42–0.47 infections per 10,000 patient days) (Table 4).

Regionally, HA-MRSA BSI rates have remained stable across all regions (Western range: 0.47–0.52; Central range: 0.37–0.52; Eastern range: 0.20–0.57; Northern range: 0.00 infections per 10,000 patient days) (Appendix B). The CA-MRSA BSI rates remained stable across all regions except for in the East where there was a significant increase by 0.41 infections per 10,000 patient days (*p*=0.027) (Western range: 0.70–0.83; Central range: 0.42–0.61; Eastern range: 0.09–0.50; Northern range: 0.00 infections per 10,000 patient days) (Appendix B). In 2023, CA-MRSA BSI rates were highest in Western Canada (0.77 infections per 10,000 patient days), and HA-MRSA BSI rates were highest in Eastern Canada (0.57 infections per 10,000 patient days) (Appendix B).

**Hospital types:** Both HA- and CA-MRSA BSI rates were consistently higher from 2019 to 2023 in adult (HA-MRSA range: 0.44–0.54, *p*=0.007; CA-MRSA range: 0.57–0.71, *p*<0.001) and mixed hospitals (HA-MRSA range: 0.38–0.48, *p*=0.01; CA-MRSA range: 0.54–0.78, *p*=0.004), with lower rates observed in paediatric hospitals (HA-MRSA range: 0.24–0.41; CA-MRSA range: 0.28–0.36 infections per 10,000 patient days) (Appendix B). Stratified by hospital size, both HA-and CA-MRSA BSI rates were generally highest among medium (201–499 beds; HA-MRSA *p*=0.02; CA-MRSA *p*<0.001) and large size hospitals (500 or more beds; HA-MRSA *p*=0.07; CA-MRSA *p*<0.001) (Appendix B).

**30-day all-cause mortality:** Thirty day all-cause mortality remained stable from 2019 to 2023 (range: 16.4–19.9) (Table 4). In 2023, 30-day all-cause mortality was significantly higher for HA-MRSA (24.7%) compared to CA-MRSA (15.2%) (*p*<0.001).

**Antimicrobial resistance:** Clindamycin resistance among MRSA isolates decreased significantly from 40% to 20% between 2019 and 2023 (*p*=0.027) (**Table 5**). Since 2019, the proportion of MRSA isolates resistant to erythromycin and ciprofloxacin has stayed relatively stable and high at around 70% in relation to other antibiotics tested. All tested MRSA BSI isolates from 2019 to 2023 were susceptible to linezolid, daptomycin and vancomycin.

**Table 5:** Methicillin-resistant *Staphylococcus aureus* bloodstream antimicrobial resistance data, Canada, 2019–2023^a^

Antibiotic	Year									
	2019		2020		2021		2022		2023	
	n	%	n	%	n	%	n	%	n	%
Ciprofloxacin	561	70.5	460	65.6	491	65.9	418	66.3	466	67.1
Clindamycin	297	37.3	234	33.4	221	29.7	159	25.2	161	23.2
Daptomycin	0	0	0	0	0	0	0	0	0	0
Erythromycin	603	75.8	507	72.3	510	68.5	431	68.4	487	70.1
Gentamicin	35	4.4	22	3.1	36	4.8	20	3.2	29	4.2
Linezolid	1	0.1	0	0	0	0	0	0	0	0
Rifampin	7	0.9	6	0.9	10	1.3	5	0.8	8	1.2
Trimethoprim/sulfamethoxazole	62	7.8	46	6.6	64	8.6	52	8.3	67	9.6
Tetracycline	0	0.0	1	0.1	6	0.8	5	0.8	4	0.6
Tigecycline	15	1.9	16	2.3	32	4.3	37	5.9	18	2.6
Vancomycin	0	0	0	0	0	0	0	0	0	0
Total number of isolates tested^b,c^	796	N/A	701	N/A	745	N/A	630	N/A	695	N/A

Abbreviation: N/A, not applicable

^a^ All MRSA isolates from 2019 to 2023 submitted to the National Microbiology Laboratory were susceptible to nitrofurantoin

^b^ In some years, the number of isolates tested for resistance varied by antibiotic

^c^ Total reflects the number of isolates tested for each of the antibiotics listed above

Comparing isolates from HA-MRSA with CA-MRSA cases, clindamycin resistance was consistently higher among isolates from HA-MRSA each year from 2019 (47.5%, n=160/337 vs. 30.3%, n=122/403) to 2023 (31.6%, n=87/275 vs. 17.0%, n=70/412) (Appendix B). There were no other notable differences in antibiotic resistance patterns by MRSA BSI case type.

**Molecular typing:** Between 2019 and 2023, the proportion of spa types identified as t002, most commonly associated with HA-MRSA, continued to decrease from 20.2% of all isolates in HA-MRSA cases in 2019 to 9.5% in 2023 (*p*<0.001) (Appendix B). Meanwhile, spa type t008, historically most commonly associated with CA-MRSA, continued to increase and account for the largest proportion of isolates identified in CA-MRSA (42.4% in 2019 to 48.8% in 2023, *p*=0.04) and HA-MRSA defined cases (28.5% in 2019 to 40.0% in 2023, *p*<0.001) (Appendix B).

### Vancomycin-resistant *Enterococcus* bloodstream infections

From 2019 to 2023, VRE BSI rates significantly increased from 0.30 to 0.37 infections per 10,000 patient days (*p*=0.02) (**Table 6**). The median age among patients with VRE BSI was 62 years (IQR: 51–71) and women accounted for 40% of VRE BSI cases (Appendix B).

**Table 6:** Vancomycin-resistant *Enterococcus* bloodstream infections data, Canada, 2019–2023^a^

VRE BSI data	Year									
2019		2020		2021		2022		2023	
**Vancomycin-resistant *Enterococcus* bloodstream infections data**										
Number of VRE BSIs	241		224		251		305		318	
Rate per 1,000 patient admissions	0.23		0.23		0.24		0.29		0.29	
Rate per 10,000 patient days	0.30		0.30		0.32		0.36		0.37	
Number of reporting hospitals	70		81		80		80		84	
**All-cause mortality rate^b^**										
Number of deaths	83		82		84		117		117	
All-cause mortality rate per 100 cases	34.4		36.6		33.5		38.4		36.8	
**Antimicrobial resistance of *Enterococcus faecium* isolates**	**n**	**%**	**n**	**%**	**n**	**%**	**n**	**%**	**n**	**%**
Ampicillin	173	100	132	98.5	166	98.8	199	97.5	216	97.7
Chloramphenicol	30	17.3	28	20.9	51	30.4	34	16.7	36	16.3
Ciprofloxacin	173	100	132	98.5	166	98.8	203	99.5	219	99.1
Daptomycin^c^	7	4.0	6	4.5	5	3.0	4	2.0	4	1.8
Erythromycin	166	96.0	128	95.5	159	94.6	199	97.5	214	96.8
High-level gentamicin	57	32.9	36	26.9	34	20.2	39	19.1	40	18.1
Levofloxacin	173	100	131	97.8	166	98.8	202	99.0	219	99.1
Linezolid	3	1.7	1	0.7	3	1.8	6	2.9	1	0.5
Nitrofurantoin	66	38.2	56	41.8	131	78.0	143	70.1	136	61.5
Penicillin	173	100	133	99.3	166	98.8	200	98.0	216	97.7
Quinupristin/dalfopristin	18	10.4	9	6.7	8	4.8	16	7.8	34	15.4
Rifampicin	160	92.5	115	85.8	155	92.3	188	92.2	204	92.3
High-level streptomycin	42	24.3	29	21.6	48	28.6	51	25.0	62	28.1
Tetracycline	119	68.8	89	66.4	134	79.8	180	88.2	180	81.4
Tigecycline	0	0	0	0	0	0	0	0	0	0
Vancomycin	170	98.3	130	97.0	163	97.0	203	99.5	221	100
Total number of isolates tested^d^	173	N/A	134	N/A	168	N/A	204	N/A	221	N/A

Abbreviations: N/A, not applicable; VRE BSI, vancomycin-resistant *Enterococcus* bloodstream infection

^a^ Due to the low number of CA-VRE BSI cases reported each year, this table presents data for all cases combined (HA and CA)

^b^ Based on the number of cases with associated 30-day outcome data

^c^ Clinical and Laboratory Standards Institute (CLSI) resistance breakpoints came into effect in 2024 and was applied to all years (CLSI M100 ED34:2024)

^d^ Total reflects the number of isolates tested for each of the antibiotics listed above

Note: Aggregate mortality data reported in-text due to fluctuations in the small numbers of VRE BSI deaths reported each year

Note: Antimicrobials presented are for surveillance purposes. Please refer to CLSI for appropriate treatment of BSI *Enterococcus* infections (CLSI M100 ED34:2024)

**Source of infection:** Vancomycin-resistant *Enterococcus* BSIs were predominantly HA, as 89.5% (n=1,199/1,339) of VRE BSIs reported from 2019 to 2023 were acquired in a healthcare facility. Stratified by source of infection, HA-VRE BSI rates significantly increased from 2019 to 2023 from 0.27 to 0.33 infections per 10,000 patient days (*p*=0.03) (Appendix B). CA-VRE BSI rates remained low and stable over time (range: 0.02–0.04 infections per 10,000 patient days).

Regionally, VRE BSI rates in Western Canada significantly increased from 0.29 to 0.48 infections per 10,000 patient days from 2019 to 2023 (*p*=0.04). No significant trend was observed in Central (range: 0.29–0.39 infections per 10,000 patient days, *p*=1.00) and Eastern Canada (range: 0–0.04 infections per 10,000 patient days, *p*=0.16) (Appendix B).

**Hospital types:** Stratified by hospital type, VRE BSI rates remained highest in adult hospitals from 2019 to 2023 (range: 0.38–0.48 infections per 10,000 patient days). From 2019 to 2023, VRE BSI rates in paediatric hospitals were low (range: 0–0.25 infections per 10,000 patient days). In 2019, VRE BSI rates were highest in small (1–200 beds) and large hospitals (500 or more beds) at 0.35 infections per 10,000 patient days compared to 0.26 infections per 10,000 patient days in medium hospitals (201–499 beds). No significant trend was observed over time across all categories of hospital bed sizes. In 2023, VRE BSI rates in large hospitals were highest at 0.49 infections per 10,000 patient days compared to 0.31 in medium hospitals and 0.16 in small hospitals (Appendix B). The incidence rates for HA-VRE BSI by region, hospital type and hospital size are presented in Appendix B.

**30-day all-cause mortality:** All-cause mortality remained high and stable over time from 2019 to 2023 (range: 33.5–38.4) (*p*=0.23) (Table 6).

**Antimicrobial resistance:** Between 2019 to 2023, high-level gentamicin resistance among VRE BSI isolates (*E. faecium*) significantly decreased from 32.9% to 18.1% (*p*=0.01) (Table 6). Daptomycin resistance, significantly decreased from 4.0% (n=7 isolates) in 2019 to 1.8% (n=4 isolates) in 2023 (*p*=0.04).

**Molecular typing:** From 2019 to 2023, the majority of VRE BSI isolates were identified as *E. faecium*; however, one *E. faecalis* was identified in 2020 (0.7%), 2021 (0.6%) and 2022 (0.5%), respectively, and three (1.4%) in 2023 (Appendix B). The increased presence of VanB among *E. faecium* changed from 0.6% (n=1) in 2019 to 7.2% (n=16) in 2023 (Appendix B). Among *E. faecium* isolates, a shift in predominant sequence types was observed over the past five years. The proportion identified as sequence type (ST)1478 was highest in 2019 (31.2%, n=54/173) and significantly decreased to 1.4% (n=3/221) in 2023 (*p*=0.04) (Appendix B). The proportion of ST17 isolates increased non-significantly from 2019 (17.9% n=31/173) to 2023 (30.3%, n=67/221) (*p*=0.40) (Appendix B). The proportion of ST80 isolates increased significantly from 2019 (15.6%, n=27/173) to 2023 (31.7%, n=70/221) (*p*=0.01) (Appendix B) and now represents the predominant ST amongst all tested isolates.

### Carbapenemase-producing *Enterobacterales* (CPE) and *Acinetobacter baumannii* (CPA)

From 2019 to 2023, CPE infection rates have remained low compared to other HAIs in Canada, although there has been a non-significant increase in the rates over this period (0.08–0.16 infections per 10,000 patient days, *p*=0.08) (**Table 7**). The number of CPA infections were very low with five or fewer cases per year between 2019 and 2023. The median age for CPE infections was 65 years and 43% of cases were female (Appendix B).

**Table 7:** Carbapenemase-producing *Enterobacterales* data, Canada, 2019–2023

CPE data	Year									
	2019		2020		2021		2022		2023	
**Number of infections and incidence rates**										
Number of CPE infections	56		40		77		111		162	
Infection rate per 1,000 patient admissions	0.06		0.04		0.08		0.10		0.13	
Infection rate per 10,000 patient days	0.08		0.06		0.10		0.13		0.16	
Number of reporting hospitals	66		81		81		85		97	
**All-cause mortality rate**										
Number of CPE infection deaths	12		7		15		17		24	
All-cause mortality rate per 100 cases	27.3		17.5		19.7		18.9		16.4	
**Carbapenemases identified^a^**	**n**	**%**	**n**	**%**	**n**	**%**	**n**	**%**	**n**	**%**
KPC	131	42.4	98	40	178	50.1	214	45.3	319	34.9
NDM	104	33.7	80	32.7	85	23.9	131	27.8	317	34.7
OXA-48	46	14.9	48	19.6	57	16.1	94	19.9	189	20.7
SME^b^	1	0.3	2	0.8	1	0.3	0	0	1	0.1
NDM/OXA-48	16	5.2	9	3.7	12	3.4	14	3	52	5.7
GES	1	0.3	0	0	1	0.3	0	0	0	0
IMP	1	0.3	1	0.4	2	0.6	2	0.4	1	0.1
NMC	4	1.3	7	2.9	15	4.2	3	0.6	12	1.3
VIM	3	1	0	0	1	0.3	6	1.3	4	0.4
Other	2	0.6	0	0	3	0.8	8	1.7	18	2
Total number of isolates tested^c^	309	N/A	245	N/A	355	N/A	472	N/A	913	N/A

Abbreviations: CPE, carbapenemase-producing *Enterobacterales*; GES, Guiana extended-spectrum β-lactamase; IMP, active-on-imipenem; KPC, *Klebsiella pneumoniae*; carbapenemase; NDM, New Delhi metallo-β-lactamase; NMC, not metalloenzyme carbapenemase; N/A, not applicable; OXA-48, oxacillinase-48; SME, *Serratia marcescens* enzymes; VIM, Verona integron-encoded metallo-β-lactamase

^a^ Includes data for all CPE isolates submitted (infections and colonisations)

^b^ Only found in *Serratia marcescens*

^c^ Some isolates contain multiple carbapenemases therefore the total number of isolates tested and the number of carbapenemases indicated may not match. *Acinetobacter baumanii* were not included in this table

Note: All-cause mortality only includes CPE infections that have a 30-day outcome available

From 2019 to 2023, the majority of CPE infections (94.8%) were almost equally distributed between Central (49.3%, n=220/446) and Western Canada (45.5%, n=203/446) while few infections were identified in the East (5.2%, n=23/446) (Appendix B). From 2019 to 2023, large hospitals (500 or more beds) generally reported the highest rates of CPE infections (0.09–0.22 infections per 10,000 patient days) compared to small hospitals (fewer than 200 beds) (0.1–0.07 infections per 10,000 patient days. During this period, 30.8% (n=102/331) of CPE-infected patients reported travel outside of Canada and of those, 83.3% (n=75/90) received medical care while abroad. The majority of CPE infections were acquired domestically with 84.2% (n=331/393) of CPE infections acquired in Canada and 81.9% (n=271/331) acquired within a Canadian acute care hospital between 2019 and 2023.

**Organisms:** Of all isolates submitted (infections and colonizations), the top four carbapenemase producing organisms during 2023 were *Escherichia coli* (41.3%), *Klebsiella pneumoniae* (16.4%), *Enterobacter cloacae* (16.4%) and *Citrobacter freundii* (14.2%). From 2019 to 2023, there has been an increase in the proportion of *E. coli*-producing carbapenemases (33%–41.3%) and a decrease in the proportion of *K. pneumoniae-* (21.4%–16.4%) and *E. cloacae-* (19.8%–16.4%) producing carbapenemases (Appendix B). The predominant carbapenemases, in order identified in Canada, were *K. pneumoniae* carbapenemase (KPC), New Delhi metallo-β-lactamase (NDM) and oxacillinase-48 (OXA-48), accounting for 96.2% to 96.0% of identified carbapenemases from 2019 to 2023. Historically, KPC has been the most commonly identified carbapenemase in Canada; however, the proportion of KPC and NDM have been continually trending closer and were almost equal in 2023.

**30-day all-cause mortality:** All-cause mortality for CPE infections fluctuated between 2019 and 2023 with a mean of 20% (Table 7).

**Antibiotic resistance:** Multidrug resistance (MDR) and extensive drug resistance (XDR) was observed among CPE (Appendix B) ((21)). In all years, NDM producing isolates were predominantly XDR (range: 83.8–91.8). Conversely, in 2019, OXA-48-like producers were previously associated with a higher proportion of XDR or MDR (89.1%) compared to 2023; (63.5%) showing an overall downward trend in resistance. *Klebsiella pneumoniae* carbapenemase has been more equally distributed throughout 2019–2023 for either XDR (range: 40.8–50.1) or MDR (range: 40.8–52.2). When examining resistance among the top three carbapenemases, we noted that there was an increase in resistance to all aminoglycosides from 2021 to 2023 in KPC producers (Appendix B). Conversely, among OXA-48-like producers, there was a decline in resistance to aztreonam, doxycycline, levofloxacin, minocycline, trimethoprim/sulfamethoxazole, carbapenems, cephalosporins, tobramycin and gentamicin. This agrees with observations that less OXA-48-like producers were XDR or MDR over time. From 2019 to 2023, the overall resistance in KPC, NDM and OXA-48-like producers to ertapenem was 78.2%, 97.8% and 66.9%, respectively, and for meropenem was 59.1%, 92.3% and 16.5%. respectively. Among new combination drugs, KPC and OXA-48-like producers were highly susceptible to meropenem/vaborbactam and ceftazidime/avibactam. Resistance to imipenem/relebactam by year ranged from 11.1%–17.4% in OXA-48-like producers and 86.3%–93.1% in NDM producers. Meropenem/vaborbactam resistance in NDM producers ranged from 61.3%–76% by year.

### Candida auris

Sixty-six percent (n=72/109) of CNISP hospitals participate in *C. auris* surveillance and between CNISP and the National Microbiology Laboratory surveillance, a total of 36 isolates (colonizations and infections) have been reported from 2019 to 2023. The number of *C. auris* cases detected per year was seven in 2019, four in 2020, three in 2021, 12 in 2022 and 10 in 2023. Fourteen cases were from Western Canada, 20 cases were from Central Canada and two cases were reported from Eastern Canada. Of the 36 *C. auris* isolates, 19.4% were resistant to amphotericin B and 77.8% were resistant to fluconazole (**Table 8**). The amphotericin B resistant isolates were also fluconazole resistant, thus 19.4% of isolates were multidrug-resistant (resistant to two classes of antifungals). Based on available travel information, 73.3% of those reporting travel also received healthcare abroad (Table 8). Of the eleven patients who received healthcare abroad, seven had known carbapenemase-producing organism (CPO) status and two were CPO positive.

**Table 8:** Antifungal resistance of *Candida auris* isolates, Canada, 2019–2023

Isolate or patient characteristics	Number of cases (n=36)		
	n		%
**Antifungal resistance of *Candida auris* isolates**			
Fluconazole	28	77.8	
Amphotericin B	7	19.4	
Fluconazole and amphotericin B (multidrug resistance)	7	19.4	
Micafungin	0	0	
**Travel history**			
Receipt of health care abroad	11	73.3	
Travel abroad (no health care reported)	1	6.7	
No travel reported	3	20	
Unknown travel history	21	N/A	

Abbreviation: N/A, not applicable

## Discussion

Canadian Nosocomial Infection Surveillance Program data have shown that between 2019 and 2023, infection rates in Canada have remained relatively stable for CDI (3%) and MRSA BSI (−0.8%). Rates have increased for VRE BSI and CPE infections (23.3% and 100%, respectively), but remain lower than CDI and MRSA BSI rates. A total of 36 *C. auris* isolates were identified from 2019 to 2023.

The MRSA BSI patients had a median age of 55 years (IQR: 39–70) and were younger compared to those with CDI (69 years) or VRE BSI (62 years) cases. The median time from admission to a positive test for HA-MRSA BSI patients related to your acute care facility was 13 days (IQR: 3–30), which was shorter than for VRE BSI (19 days) and CPE (20 days) but longer than CDI (10 days). The CDI infections occurred more equally between males and females (50% each), compared to 40% females for VRE BSI infections, 38% females for MRSA BSI infections and 43% females for CPE infections (Appendix B).

Trends in CDI rates observed in the CNISP network align with similar trends reported globally ((22)) where COVID-19 may have contributed to the increase in 2020 of both HA and CA-CDI rates following pre-COVID-19 pandemic declines ((23)). Beyond COVID-19, HA-CDI rates continued to decline while CA-CDI rates returned to pre-pandemic levels ((23)). When comparing globally, both HA- (3.85 per 10,000 patient days) and CA-CDI (1.83 per 1,000 patient admissions) rates observed in the CNISP network were higher than those reported in acute care hospitals in European Union/European Economic Area countries, which reported an HA-CDI rate of 2.58 per 10,000 patient days and a CA-CDI rate of 1.35 per 1,000 patient admissions in 2020 ((22)).

*Clostridioides difficile* antimicrobial resistance is less common in Canada than in the US or globally ((24)). In a representative sample of Canadian acute care hospitals, from 2019 to 2023, a 5.3% decrease in moxifloxacin resistance in both HA- and CA-CDI populations is concordant with an overall decrease in the prevalence of RT027. Furthermore, moxifloxacin resistance remained lower (6.3% in 2023) than previously published weighted pooled resistance data for North America (44.0%) and Asia (33.0%) ((25,26)). The decline in the prevalence of RT027 has been replaced with a concomitant increase in the prevalence of RT106, RT014 and RT020, consistent with trends observed in the US ((27,28)). Additionally, the emergence of RT106 now found worldwide, presents additional challenges as this strain has been shown to produce more spores, have higher rates of recurrence, and is highly resistant to erythromycin, clindamycin, fluoroquinolones and third-generation cephalosporins. The potential emergence of resistant ribotypes warrants further surveillance, monitoring and investigation ((27,29)).

Between 2019 and 2023, MRSA BSI rates in the CNISP network remained stable, fluctuating between 1.00 to 1.16 infections per 10,000 patient days. From 2019 to 2023, HA-MRSA BSI rates in CNISP (0.42–0.47 infections per 10,000 patient days), were notably higher than rates reported in Australian public hospitals between 2018 and 2022 (0.11–0.13 infections per 10,000 patient days), likely due to broader CNISP definitions that capture more cases with indirect healthcare links ((30)). However, the CNISP rate for 2023 (0.47 infections) is similar to the rate reported in US hospitals for the same year (0.49 infections per 10,000 patient days), where definitions for laboratory-based surveillance are similar ((31)). The CA-MRSA BSI rate in CNISP for 2023 (0.67 infections per 10,000 patient days) is lower than the rate reported in US hospitals for the same year (0.84 infections), reflective of different populations ((31)). Community-associated-MRSA BSI rates have shown a sustained increase in CNISP data since 2019, suggesting an expanding community reservoir of MRSA in Canada and globally ((32,33)).

The CNISP 30-day all-cause mortality rates for MRSA BSI (HA: 20.1%–25.0%; CA: 13.6%–17.4%) were lower than those reported in the US (HA: 29%; CA: 18%) ((33)). Differences may stem from CNISP’s strict 30-day mortality cut-off versus undefined US time frames, or from variances in healthcare systems, infection prevention strategies and population characteristics ((34,35)).

A significant 20% decrease in clindamycin resistance among MRSA BSI isolates between 2019 and 2023 coincided with shifts in MRSA spa types. The proportion of spa type t002 (commonly HA-MRSA) declined, while spa type t008 (historically CA-MRSA) increased. Notably, t008 rose among CA-MRSA isolates (42.4% to 48.8%) and HA-MRSA isolates (28.5% to 40.0%). This shift underscores the increasing role of CA-MRSA clones in healthcare settings and highlights the dynamic nature of MRSA epidemiology. The growing prevalence of traditionally CA clones in hospitals emphasizes the need for ongoing surveillance and tailored infection prevention strategies. Continued monitoring of antimicrobial resistance patterns is critical for guiding treatment protocols and mitigating MRSA burden in healthcare and community settings. Populations at heightened risk for CA-MRSA infection include children, athletes, incarcerated individuals, seniors with comorbidities and people who inject drugs ((34,35)). Injection drug use in particular may signal the emergence of an at-risk population for CA-MRSA. Strategies such as screening and decolonization of MRSA carriers in high-risk populations could help reduce the overall burden of MRSA BSIs ((34–36)).

Vancomycin resistance related to VRE BSI has been shown to be associated with higher mortality rates and longer hospital stays, making it a significant public health concern ((37–39)). Vancomycin-resistant *Enterococcus* BSI rates observed in the CNISP network increased over time between 2019 and 2023 and were highest in 2023 (0.37 infections per 10,000 patient days). The success of certain sequence types likely contributes to the increased burden of VRE BSI in CNISP-participating hospitals. As of 2023, ST17 (30.3%) and ST80 (31.7%) were the predominant clones overtaking the previously dominant clone ST1478 (1.4%). Compared to other sequence types, a distinct association has been identified between ST80 and the VanB gene. This association of VanB genes harboured predominantly among ST80 isolates has also been documented in recent studies related to VanB outbreaks in Sweden and Denmark ((40,41)). The VRE BSI trends are further impacted by the number of high-risk patients admitted to hospital (e.g., bone marrow transplants, solid organ transplants, cancer patients, etc.) ((42,43)). Most VRE BSI cases reported by CNISP-participating hospitals were healthcare-acquired, highlighting the importance of appropriate screening, adherence to infection prevention measures and antimicrobial stewardship. Although there is a lack of recent data on VRE BSI rates in comparable jurisdictions, there have been increasing trends noted in Europe ((44–48)), which may be associated, in part, with the introduction and spread of new clones and gaps in infection prevention practices ((44,45,49)).

Carbapenemase-producing *Enterobacterales* infections are a significant threat to public health as they are becoming increasingly prevalent in healthcare environments worldwide, are associated with high mortality and limited treatment options ((50–53)). The Centers for Disease Control and Prevention and the World Health Organization have classified CPE as one of the most urgent antimicrobial-resistance threats ((54,55)). While the number of CPE infections doubled from 2019 to 2023 in the CNISP network, incidence remained low compared to other HAIs. Data on the incidence of CPE infections in other countries, such as Denmark, Italy, Switzerland and the United Kingdom, have also shown an increasing incidence of CPE infections ((56–59)). Historically, CPE infections were mostly associated with international travel, but there has been a shift in recent years to domestic acquisition. From 2020 to 2023, 84.6% of CPE infections were domestically acquired and 80.8% were acquired in a Canadian acute care hospital, suggesting that within hospital transmission is driving the recent increase in CPE infection incidence. As a result, strict implementation of infection control measures, including screening in patients with a previous hospital admission domestically and abroad, are useful to reduce the transmission of CPE in Canadian acute care hospitals.

*Candida auris* is an emerging multidrug resistant fungus that can cause HA invasive infections and outbreaks ((60)). It has been detected across multiple countries and continents including Canada, since its first detection in 2009 ((61–64)). *Candida auris* has been associated with outbreaks in healthcare settings in many countries, including Canada and the US, although outbreaks in Canada to date have been limited with few cases ((60)). Reported crude mortality for *C. auris* ranges widely from 15%–60% but is generally similar to other *Candida* species ((60–66)). Though still relatively rare in Canada, the US reported over 4,500 clinical cases and over 9,000 screening cases in 2023 ((67)). The identification of *C. auris* in routine microbiology laboratories requires identification of *Candida* to the species level, which may not be routinely performed for isolates from non-sterile sites. Treatment options are limited for patients as approximately one-third of identified *C. auris* isolates in Canada were multidrug-resistant and additional resistance can develop during antifungal therapy ((68)). Therefore, rapid identification, screening for colonization in at-risk patients and strict implementation of infection prevention and control measures are required to reduce the transmission of *C. auris* in Canadian healthcare settings. Continued reporting on *C. auris* in Canada is important to assess and monitor the risk of this pathogen, in addition to identifying epidemiological and microbiological trends ((69)).

### Strengths and limitations

The main strength of CNISP is the collection of standardized and detailed epidemiological and laboratory-linked data from 109 sentinel hospitals across Canada for the purpose of providing national HAI and AMR trends for benchmarking and to guide hospital infection prevention and control practices.

Epidemiological data collected by CNISP were limited to information available in-patient charts. Hospital staff turnover may affect the consistent application of CNISP definitions when reviewing medical charts; however, these data were collected by experienced and trained infection prevention and control staff who receive periodic training with respect to CNISP methods and definitions. Furthermore, data quality assessments were conducted to maintain and improve data quality. These data may be subject to potential selection bias due to the exclusion of sites with missing or incomplete data throughout the study period. A limitation of *C. auris* surveillance is that detailed epidemiologic data are only available on patients identified at CNISP participating hospitals. From 2019 to 2023, CNISP coverage of Canadian acute care beds has increased from 33% to 37%, including increased representativeness in northern, community, rural and Indigenous populations.

## Conclusion

Surveillance findings from a national sentinel network of Canadian acute care hospitals indicate that rates of MRSA BSI and CDI have remained stable from 2019 to 2023, while rates of VRE BSI and CPE infections have increased. Few cases of *C. auris* were detected in Canada. Consistent and standardized surveillance of epidemiologic and laboratory HAI data are essential to providing hospital practitioners with benchmark rates and informing infection prevention and control and antimicrobial stewardship policies to help reduce the burden of HAI and the impact of AMR in Canadian acute care hospitals.

Efforts to improve the quality and representativeness of Canadian HAI surveillance data are ongoing. The enhanced hospital screening practices survey is conducted annually to better understand and contextualize changes in HAI rates in the CNISP network. In addition, CNISP conducts point prevalence survey (PPS) to assess the burden and incidence of HAIs and antimicrobial use in participating Canadian acute care hospitals, and to establish ongoing benchmark rates. CNISP’s fourth PPS was conducted from February to March 2024. The CNISP continues to update HAI, antibiotic-resistant organism rates and viral respiratory infection rates, including COVID-19, on a publicly available dashboard using Canada’s Health Infobase ((70)). To further improve representativeness and generalizability of national HAI benchmark rates, CNISP has launched a simplified dataset accessible to all acute care hospitals across Canada to collect and visualize annual HAI rate data and has over 100 hospitals participating in the project. Finally, CNISP is exploring HAI surveillance in the long-term care sector in Canada to better understand the burden of HAIs among this at-risk population.

## Appendix

### Appendix A: Surveillance case definitions and eligibility criteria, 2023

#### *Clostridioides difficile* infection

A “primary” episode of *Clostridioides difficile* infection (CDI) is defined either as the first episode of CDI ever experienced by the patient or a new episode of CDI that occurs greater than eight weeks after the diagnosis of a previous episode in the same patient.

A patient is identified as having CDI if:

• The patient has diarrhea or fever, abdominal pain and/or ileus AND a laboratory confirmation of a positive toxin assay or positive polymerase chain reaction (PCR) test for *C. difficile* (without reasonable evidence of another cause of diarrhea)

OR

• The patient has a diagnosis of pseudomembranes on sigmoidoscopy or colonoscopy (or after colectomy) or histological/pathological diagnosis of CDI

OR

• The patient is diagnosed with toxic megacolon (in adult patients only)

Diarrhea is defined as one of the following:

• More watery/unformed stools in a 36-hour period

OR

• More watery/unformed stools in a 24-hour period and this is new or unusual for these patient (in adult patients only)

Exclusion:

• Any patients aged younger than one year

• Any paediatric patients (aged one year to younger than 18 years) with alternate cause of diarrhea found (i.e., rotavirus, norovirus, enema or medication, etc.) are excluded even if the *C. difficile* diagnostic test result is positive

#### *Clostridioides difficile* infection case classification

Once a patient has been identified with CDI, the infection will be classified further based on the following criteria and the best clinical judgment of the healthcare and/or infection prevention and control practitioner.

Healthcare-associated (acquired in your facility) CDI case definition:

• Related to the current hospitalization:

o The patient’s CDI symptoms occur in your healthcare facility three or more days (or 72 hours or longer) after admission

• Related to a previous hospitalization:

o Inpatient: the patient’s CDI symptoms occur less than three days after the current admission (or fewer than 72 hours) AND the patient had been previously hospitalized at your healthcare facility and discharged within the previous four weeks

o Outpatient: the patient presents with CDI symptoms at your emergency room (ER) or outpatient location AND the patient had been previously hospitalized at your healthcare facility and discharged within the previous four weeks

• Related to a previous healthcare exposure at your facility:

o Inpatient: the patient’s CDI symptoms occur less than three days after the current admission (or fewer than 72 hours) AND the patient had a previous healthcare exposure at your facility within the previous four weeks

o Outpatient: the patient presents with CDI symptoms at your ER or outpatient location AND the patient had a previous healthcare exposure at your facility within the previous four weeks

Healthcare-associated (acquired in any other healthcare facility) CDI case definition:

• Related to a previous hospitalization at any other healthcare facility:

o Inpatient: the patient’s CDI symptoms occur less than three days after the current admission (or fewer than 72 hours) AND the patient is known to have been previously hospitalized at any other healthcare facility and discharged/transferred within the previous four weeks

o Outpatient: the patient presents with of CDI symptoms at your ER or outpatient location AND the patient is known to have been previously hospitalized at any other healthcare facility and discharged/transferred within the previous four weeks

• Related to a previous healthcare exposure at any other healthcare facility:

o Inpatient: the patient’s CDI symptoms occur less than three days after the current admission (or fewer than 72 hours) AND the patient is known to have had a previous healthcare exposure at any other healthcare facility within the previous four weeks

o Outpatient: the patient presents with CDI symptoms at your ER or outpatient location AND the patient is known to have had a previous healthcare exposure at any other healthcare facility within the previous four weeks

Healthcare-associated CDI but unable to determine which facility:

The patient with CDI DOES meet both definitions of healthcare-associated (acquired in your facility) AND healthcare-associated (acquired in any other healthcare facility) CDI, but unable to determine to which facility the case is primarily attributable to.

Community-associated CDI case definition:

• Inpatient: the patient’s CDI symptoms occur less than three days (or fewer than 72 hours) after admission, with no history of hospitalization or any other healthcare exposure within the previous 12 weeks

• Outpatient: the patient presents with CDI symptoms at your ER or outpatient location with no history of hospitalization or any other healthcare exposure within the previous 12 weeks

Indeterminate CDI case definition:

The patient with CDI does NOT meet any of the definitions listed above for healthcare-associated or community-associated CDI. The symptom onset was more than four weeks but fewer than 12 weeks after the patient was discharged from any healthcare facility or after the patient had any other healthcare exposure.

#### Methicillin-resistant *Staphylococcus aureus* (MRSA) infection

MRSA bloodstream infection (BSI) case definition:

• Isolation of *Staphylococcus aureus* from blood

AND

• Patient must be admitted to the hospital

AND

• Is a “newly identified *S. aureus* infection” at a Canadian Nosocomial Infection Surveillance Program (CNISP) hospital at the time of hospital admission or identified during hospitalization

Infection inclusion criteria:

• Methicillin-susceptible *Staphylococcus aureus* (MSSA) or MRSA BSIs identified for the first time during this current hospital admission

• MSSA or MRSA BSIs that have already been identified at your site or another CNISP site but are **new** infections

Criteria to determine NEW MSSA or MRSA BSI:

• Once the patient has been identified with a MSSA or MRSA BSI, they will be classified as a new MSSA or MRSA if they meet the following criteria: more than 14 days since previously treated MSSA or MRSA BSI and, in the judgment of infection control physicians and practitioners, represents a new infection

Infection exclusion criteria:

• Emergency, clinic, or other outpatient cases who are **NOT admitted** to the hospital

Healthcare-associated (HA) case definition:

Healthcare-associated is defined as an inpatient who meets the following criteria and in accordance with the best clinical judgment of the healthcare and/or infection prevention and control practitioner:

• Patient is on or beyond calendar day 3 of their hospitalization (calendar day 1 is the day of hospital admission)

OR

• Has been hospitalized in your facility in the last 7 days or up to 90 days depending on the source of the infection

OR

• Has had a healthcare exposure at your facility that would have resulted in this bacteremia (using best clinical judgment)

OR

• Any patient who has a bacteremia not acquired at your facility that is thought to be associated with any other healthcare exposure (e.g., another acute-care facility, long-term care, rehabilitation facility, clinic or exposure to a medical device)

Healthcare-associated (HA) case definition (newborn):

• The newborn is on or beyond calendar day 3 of their hospitalization (calendar day 1 is the day of hospital admission)

• The mother was **NOT** known to have MRSA on admission and there is no epidemiological reason to suspect that the mother was colonized prior to admission, even if the newborn is fewer than 48 hours of age

• In the case of a newborn transferred from another institution, MSSA or MRSA BSI may be classified as HA your acute-care facility if the organism was **NOT** known to be present and there is no epidemiological reason to suspect that acquisition occurred prior to transfer

Community-associated case definition:

• No exposure to healthcare that would have resulted in this bacteremia (using best clinical judgment) and does not meet the criteria for a healthcare-associated BSI

#### Vancomycin-resistant *Enterococcus* (VRE) infection

VRE BSI case definition:

• Isolation of *Enterococcus faecalis* or *faecium* from blood

AND

• Vancomycin minimum inhibitory concentration (MIC) of **at least** 8 µg/ml

AND

• Patient must be admitted to the hospital

AND

• Is a “newly” identified VRE BSI at a CNISP facility at the time of hospital admission or identified during hospitalization

**A newly identified VRE BSI** is defined as a positive VRE blood isolate more than 14 days after completion of therapy for a previous infection and felt to be unrelated to previous infection in accordance with best clinical judgment by infection control physicians and practitioners.

Exclusion criteria:

• Emergency, clinic, or other outpatient cases who are **not admitted** to the hospital

Healthcare-associated (HA) case definition:

Healthcare-associated is defined as an inpatient who meets the following criteria and in accordance with the best clinical judgment of the healthcare and/or infection prevention and control practitioner:

• Patient is on or beyond calendar day 3 of their hospitalization (calendar day 1 is the day of hospital admission)

OR

• Has been hospitalized in your facility in the last 7 days or up to 90 days depending on the source of the infection

OR

• Has had a healthcare exposure at your facility that would have resulted in this bacteremia (using best clinical judgment)

OR

• Any patient who has a bacteremia not acquired at your facility that is thought to be associated with any other healthcare exposure (e.g., another acute-care facility, long-term care, rehabilitation facility, clinic or exposure to a medical device)

Community-associated case definition:

• No exposure to healthcare that would have resulted in this bacteremia (using best clinical judgment) and does not meet the criteria for a healthcare-associated BSI

#### Carbapenemase-producing *Enterobacterales* (CPE) infection

Case eligibility:

• Patient is admitted to a CNISP hospital or presents to a CNISP hospital emergency department or a CNISP hospital-based outpatient clinic

• Laboratory confirmation of carbapenem resistance or carbapenemase production in *Enterobacterales* spp.

Following molecular testing, only isolates determined to be harbouring a carbapenemase are included in surveillance. If multiple isolates are submitted for the same patient in the same surveillance year, only the isolate from the most invasive site is included in epidemiological results (e.g., rates and outcome data). However, antimicrobial susceptibility testing results represent all CPE isolates (including clinical and screening isolates from inpatients and outpatients) submitted between 2018 and 2022; duplicates (i.e., isolates from the same patient where the organism and the carbapenemase were the same) were excluded.

#### Candida auris

Patients admitted to a participating hospital or presenting to a hospital emergency department or a hospital-based outpatient clinic with laboratory confirmation of *C. auris* from any specimen.

Included in this surveillance project are all clinical or screening samples that were positive for *C. auris* by any method. Currently, *C. auris* can be identified by rRNA sequencing, Vitek MS MALDI-TOF (with either the clinical database v3.2 or later or the RUO database), or Bruker MALDI-TOF (with either the clinical database v6903 or later or the RUO database). The project also includes potential *C. auris* misidentifications or “No identification” as outlined in the **Table A1** below.

**Table A1:** Laboratory identification of *Candida auris*

Identification method	Identification of suspect isolates
Vitek MS MALDI Clinical database older than v3.2	*C. haemulonii* No ID/low discrimination *C. rugosa* (not a problem for v3.0 or later) *C. pulcherrima* (not a problem for v3.0 or later)
Bruker MALDI Clinical database older than v6903	No ID
Vitek 2 version 8.01	*C. haemulonii* *C. duobushaemulonii* No ID/low discrimination
Vitek 2 version before 8.01	*C. haemulonii* *C. duobushaemulonii* *C. lusitaniae* *C. famata* No ID/low discrimination
API 20C AUX	*Rhodotorula glutinis* (characteristic red colour not present) *C. sake* No ID/low discrimination
API Candida	*C. famata*
BD Phoenix yeast identification system	*C. haemulonii* *C. catenulata* No ID

Abbreviations: *C., Candida*; MALDI, Matrix-Assisted Laser Desorption Ionization; MS, mass spectrometry

### Appendix B

Supplemental figures and tables are available upon request to the author: cnisp-pcsin@phac-aspc.gc.ca

Table S1.0: Summary of patient characteristics for *Clostridioides difficile* infections (CDIs), carbapenemase-producing Enterobacterales (CPE) infections, methicillin-resistant *Staphylococcus aureus* (MRSA) bloodstream infections (BSIs), and vancomycin-resistant *Enterococcus* (VRE) BSIs, 2019–2023

Table S1.1: Cases and incidence rates of healthcare-associated and community-associated *Clostridioides difficile* infection by region, hospital type and hospital size, Canada, 2019–2023

Table S1.2: Antimicrobial resistance of healthcare-associated and community-associated *Clostridioides difficile* infection isolates, Canada, 2019–2023

Table S1.3: Number and proportion of common ribotypes of healthcare-associated and community-associated *Clostridioides difficile* infection cases, Canada, 2019–2023

Table S2.1: Cases and incidence rates of healthcare-associated and community-associated methicillin-resistant *Staphylococcus aureus* bloodstream infections by region, hospital type and hospital size, 2019–2023

Table S2.2: Antimicrobial resistance of healthcare-associated and community-associated methicillin-resistant *Staphylococcus aureus* bloodstream infection isolates, Canada, 2019–2023

Table S2.3: Number and proportion of select methicillin-resistant *Staphylococcus aureus* spa types (with corresponding epidemic types) identified

Table S3.1: Number of vancomycin-resistant *Enterococcus* bloodstream infections incidence rates by region, hospital type and hospital size, 2019–2023

Table S3.2: Number of healthcare-associated vancomycin-resistant *Enterococcus* bloodstream infections and incidence rates by region, hospital type and hospital size, 2019–2023

Table S3.3: Number and proportion of vancomycin-resistant *Enterococcus* bloodstream infections isolate types identified, 2019–2023

Table S3.4: Distribution of vancomycin-resistant *Enterococcus faecium* bloodstream sequence types, 2019–2023

Table S4.1: Number of carbapenemase-producing *Enterobacterales* infections and incidence rates by region, hospital type and hospital size, 2019–2023

Table S4.2: Number and proportion of main carbapenemase-producing pathogens identified

Table S4.3: Antimicrobial Susceptibility Testing for *Klebsiella pneumoniae* carbapenemase, 2019–2023

Table S4.4 Antimicrobial Susceptibility Testing for New Delhi metallo-β-lactamase, 2019–2023

Table S4.5: Antimicrobial Susceptibility Testing for OXA-48, Oxacillinase-48, 2019–2023
